# 911. Burden of Herpes Zoster among Employed Adults in the Department of Veterans Affairs Health System

**DOI:** 10.1093/ofid/ofad500.956

**Published:** 2023-11-27

**Authors:** Shaloo Gupta, Nai-Chung N Chang, Nikita Stempniewicz, Chenchu Bhavani K Tirupati, Gregorio Coronado, Julie A Lynch, Cosmina Hogea, Emily Mulvihill, Jason Gagner, Scott L DuVall

**Affiliations:** Cerner Enviza, Philadelphia, Pennsylvania; Department of Veterans Affairs Salt Lake City Health Care System, Salt Lake City, Utah; GSK, Philadelphia, Pennsylvania; Department of Veterans Affairs Salt Lake City Health Care System, Salt Lake City, Utah; Department of Veterans Affairs Salt Lake City Health Care System, Salt Lake City, Utah; Department of Veterans Affairs Salt Lake City Health Care System, University of Utah School of Medicine, Salt Lake City, Utah; GSK (currently Gilead Sciences), Philadelphia, Pennsylvania; Cerner Enviza, Philadelphia, Pennsylvania; Parexel, Salt Lake City, Utah; Department of Veterans Affairs and University of Utah School of Medicine, Salt Lake City, Utah

## Abstract

**Background:**

Herpes zoster (HZ) is characterized by a painful dermatomal rash and is associated with increased healthcare costs. Research quantifying work productivity loss and activity impairment associated with HZ is limited and outdated. This study aimed to describe severity of HZ related pain, health related quality of life, activity impairment, and work productivity among employed adults diagnosed with HZ in the Department of Veterans Affairs (VA) Health System.

**Methods:**

This cross-sectional study utilized a self-reported survey among adults in the VA health system. Eligibility was assessed biweekly from electronic health record data, and adults with an incident International Classification of Diseases, 10^th^ Revision (ICD-10) HZ diagnosis were invited to complete an online survey. Respondents were indexed on their HZ diagnosis and included if aged 18-70 years, reported working for pay at index, and had 6 months of baseline continuous enrollment. Respondents were excluded if they had a baseline HZ vaccine, did not complete the survey within 30 days after index, or reported not having HZ or experiencing a rash > 14 days prior to index. Questions used to assess outcomes were based on validated survey instruments (Table 1). Work productivity was described among respondents employed at survey completion. Data were collected from 05/04/2021 to 07/07/2022. Descriptive statistics were used to summarize survey responses.

**Figure:**
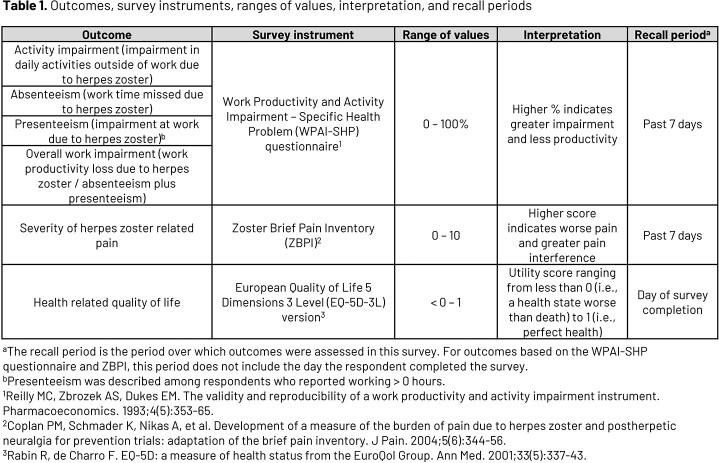

**Results:**

Among 231 respondents completing the survey, mean (standard deviation [SD]) age was 49 (10) years and 79% were male. Overall, 70% reported experiencing pain in the last 7 days. Mean (SD) [minimum, maximum] “average-pain” severity and EQ-5D-3L scores were 4.0 (2.8) [0, 10] and 0.69 (0.22) [-0.04, 1.00], respectively. Mean (SD) activity impairment was 42% (33%). Mean (SD) absenteeism, presenteeism, and overall work impairment were 17% (27%), 35% (30%), and 43% (35%) out of 202, 192, and 202 respondents, respectively.

**Conclusion:**

Burden associated with HZ is substantial, with a significant proportion experiencing pain, activity impairment, and work productivity loss. These results provide updated context on the impact of HZ in this population of employed adults in the VA health system.

**Funding:** GlaxoSmithKline Biologicals SA (GSK study identifiers: 208907/HO-16-16967).

**Disclosures:**

**Shaloo Gupta, MS; ORCID: 0000-0002-2535-2892**, Cerner Enviza: Salary|GSK: Funding to employer to conduct and support this study **Nai-Chung N. Chang, PhD; ORCID: 0000-0002-4541-8967**, Alnylam Pharmaceuticals, Inc.: Grant/Research Support|Astellas Pharma, Inc.: Grant/Research Support|AstraZeneca Pharmaceuticals LP: Grant/Research Support|Biodesix: Grant/Research Support|Celgene Corporation: Grant/Research Support|Cerner Enviza: Grant/Research Support|GSK: Grant/Research Support|Janssen Pharmaceuticals, Inc.: Grant/Research Support|Kantar Health: Grant/Research Support|Myriad Genetic Laboratories, Inc.: Grant/Research Support|Novartis International AG: Grant/Research Support|Parexel International Corporation: Grant/Research Support **Nikita Stempniewicz, MSc**, GSK: Salary|GSK: Ownership Interest **Chenchu Bhavani K. Tirupati, MS, MBA**, Alnylam Pharmaceuticals, Inc.: Grant/Research Support|Astellas Pharma, Inc.: Grant/Research Support|AstraZeneca Pharmaceuticals LP: Grant/Research Support|Biodesix: Grant/Research Support|Celgene Corporation: Grant/Research Support|Cerner Enviza: Grant/Research Support|GSK: Grant/Research Support|Janssen Pharmaceuticals, Inc.: Grant/Research Support|Kantar Health: Grant/Research Support|Myriad Genetic Laboratories, Inc.: Grant/Research Support|Novartis International AG: Grant/Research Support|Parexel International Corporation: Grant/Research Support **Gregorio Coronado, MBA**, Alnylam Pharmaceuticals, Inc.: Grant/Research Support|Astellas Pharma, Inc.: Grant/Research Support|AstraZeneca Pharmaceuticals LP: Grant/Research Support|Biodesix: Grant/Research Support|Celgene Corporation: Grant/Research Support|Cerner Enviza: Grant/Research Support|GSK: Grant/Research Support|Janssen Pharmaceuticals, Inc.: Grant/Research Support|Kantar Health: Grant/Research Support|Myriad Genetic Laboratories, Inc.: Grant/Research Support|Novartis International AG: Grant/Research Support|Parexel International Corporation: Grant/Research Support **Julie A. Lynch, PhD, RN, MBA; ORCID: 0000-0003-0108-2127**, Alnylam Pharmaceuticals, Inc.: Grant/Research Support|Astellas Pharma, Inc.: Grant/Research Support|AstraZeneca Pharmaceuticals LP: Grant/Research Support|Biodesix: Grant/Research Support|Celgene Corporation: Grant/Research Support|Cerner Enviza: Grant/Research Support|GSK: Grant/Research Support|Janssen Pharmaceuticals, Inc.: Grant/Research Support|Novartis International AG: Grant/Research Support|Parexel International Corporation: Grant/Research Support **Cosmina Hogea, PhD; ORCID: 0000-0002-0686-2395**, Gilead Sciences: Ownership Interest|GSK: Salary at the time of the submitted work|GSK: Ownership Interest **Emily Mulvihill, MBA; ORCID: 0000-0002-1430-8837**, Cerner Enviza: Salary|GSK: Funding to employer to conduct and support this study **Scott L. DuVall, PhD**, Alnylam Pharmaceuticals, Inc.: Grant/Research Support|Astellas Pharma, Inc.: Grant/Research Support|AstraZeneca Pharmaceuticals LP: Grant/Research Support|Biodesix: Grant/Research Support|Celgene Corporation: Grant/Research Support|Cerner Enviza: Grant/Research Support|GSK: Grant/Research Support|Janssen Pharmaceuticals, Inc.: Grant/Research Support|Novartis International AG: Grant/Research Support|Parexel International Corporation: Grant/Research Support

